# Defining the role of surgery for patients with multiple brain metastases

**DOI:** 10.1007/s11060-024-04739-7

**Published:** 2024-06-25

**Authors:** Tunc Faik Ersoy, Daniel Brainman, Roland Coras, Björn Berger, Florian Weissinger, Alexander Grote, Matthias Simon

**Affiliations:** 1 Department of Neurosurgery, University Hospital OWL, Campus Bielefeld-Bethel, Bielefeld, Germany; 2 Department of Neuropathology, University Hospital Erlangen, Erlangen, Germany; 3 Department for Neuroradiology, University Hospital OWL, Campus Bielefeld-Bethel, Bielefeld, Germany; 4 Department of Hematology, Oncology and Palliative Care, University Hospital OWL, Campus Bielefeld-Bethel, Bielefeld, Germany; 5 Present Address: Department of Neurosurgery, University Hospital Marburg, Marburg, Germany

**Keywords:** Multiple brain metastases, Surgery, Cytoreduction, Prognosis, Functional outcome, Complications

## Abstract

**Purpose:**

To better define the role of surgery, we investigated survival and functional outcomes in patients with multiple brain metastases.

**Methods:**

Pertinent clinical and radiological data of 131 consecutive patients (156 surgeries) were analyzed retrospectively.

**Results:**

Surgical indications included mass effect (84.6%) and need for tissue acquisition (44.9%, for molecularly informed treatment: 10 patients). Major (i.e. CTCAE grade 3–5) neurological, surgical and medical complication were observed in 6 (3.8%), 12 (7.7%), and 12 (7.7%) surgical cases. Median preoperative and discharge KPS were 80% (IQF: 60–90%). Median overall survival (mOS) was 7.4 months. However, estimated 1 and 2 year overall survival rates were 35.6% and 25.1%, respectively. Survival was dismal (i.e. mOS ≤ 2.5 months) in patients who had no postoperative radio- and systemic therapy, or who incurred major complications. Multivariate analysis with all parameters significantly correlated with survival as univariate parameters revealed female sex, oligometastases, no major new/worsened neurological deficits, and postoperative radio- and systemic therapy as independent positive prognostic parameters. Univariate positive prognostic parameters also included histology (best survival in breast cancer patients) and less than median (0.28 cm^3^) residual tumor load.

**Conclusions:**

Surgery is a reasonable therapeutic option in many patients with multiple brain metastases. Operations should primarily aim at reducing mass effect thereby preserving the patients’ functional health status which will allow for further local (radiation) and systemic therapy. Surgery for the acquisition of metastatic tissue (more recently for molecularly informed treatment) is another important surgical indication. Cytoreductive surgery may also carry a survival benefit by itself.

**Supplementary Information:**

The online version contains supplementary material available at 10.1007/s11060-024-04739-7.

## Introduction

Given the poor survival outlook and systemic affection of the CNS, the role of surgery in cases with multiple brain metastases is controversial [1–4]. The literature contains few pertinent data collected against the background of the more recent developments in medical oncology [3, 5, 6]. Generally, patients with (multiple) brain metastases are considered for surgery for the following reasons: reduction of mass effect (i.e. treatment or prevention of neurological symptoms), acquiring tissue for histological and molecular genetic analysis, and possibly cytoreduction [4, 7–9]. Beyond this, certain histologies, such as renal carcinoma or malignant melanoma are not particularly radiation-sensitive which may favor surgical therapy over irradiation in such cases [7, 10].

Large space-occupying lesions lead to neurological deterioration which may prevent otherwise necessary oncological treatment both for CNS as well as systemic disease. In the posterior fossa, such lesions may be life-threatening due to an imminent risk of occlusive hydrocephalus and brainstem compression. Reduction of the mass effect of brain metastases may therefore be vital to keep and/or render patients oncologically “treatable” [6, 11].

The role of CNS tumor load and therefore cytoreduction has also attracted some attention [12–14]. More recently, the concept of oligometastatic disease has been introduced which establishes an intermediate category between (at least for some time) presumably localized disease i.e. cases with a single metastasis and patients with multiple metastases believed to suffer from a disseminated affection of the CNS [15, 16]. Oligometastatic brain disease is often defined as suffering from two to four brain metastases [15, 17, 18]. Patients with oligometastases may benefit from therapeutic strategies aiming at local rather than systemic CNS tumor control even though several “loci” need to be addressed [4, 9, 15].

Not rarely, brain metastases are the first manifestation of the primary disease. In some cases, staging studies fail to identify a primary tumor or the presumed primary tumor is not readily accessible so that brain metastases surgery is needed for diagnostic purposes. Some tumors, such as breast cancer or malignant melanoma, show genetic discrepancies between the metastatic disease and primary tumor [19, 20]. Amid growing targeted-therapy options, acquisition of metastatic tissue may help guiding (medical) therapy (“molecularly informed treatment”) [21, 22].

The aim of the present study was to investigate the role of surgery for patients with multiple brain metastases in a large patient cohort treated in view of recent advances in medical and radio-oncology. The current literature contains only limited data on complications and functional outcomes following surgery for brain metastases [5, 6]. Can we identify patients who presumably derive a survival benefit? What is the role of surgical cytoreduction (if there is any)?

## Patients & methods

### Patients

We conducted a retrospective analysis of all consecutive patients with multiple brain metastases undergoing surgery for at least one of the tumors from January 2015 to July 2021 in our department. Cases with stereotactic biopsies only were excluded. The study was approved by the responsible institutional review board for human research and ethics committee.

All cases were discussed in the interdisciplinary neuro-oncology tumor board and additionally in disease-specific tumor boards as required. If reasonably possible, a locally aggressive approach was pursued, i.e. an attempt was made to remove all metastatic disease either by means of surgery or by combing surgery and radiosurgery [3, 23, 24].

### Surgical indications

Patients were considered as candidates for surgery when they presented with a large space-occupying tumor either already causing or believed to pose a significant risk for developing neurological deficits as long as a valid postoperative oncological therapeutic concept was available. This specifically included cases with metastases in the posterior fossa and (imminent) hydrocephalus and brainstem compression. Surgery was also performed in order to obtain tumor tissue whenever a histological diagnosis was needed and/or when primary tumor tissue was not readily accessible for diagnosis confirmation, and in some cases which required molecular studies for optimal therapeutic management. If reasonably possible, a locally aggressive approach was pursued, i.e. an attempt was made to remove all metastatic disease either by means of surgery or by combing surgery and radiosurgery, assuming that this would result in superior survival [3, 23, 24].

All cases were discussed in the interdisciplinary neuro-oncology tumor board and additionally in disease-specific tumor boards as required. Neuronavigation and in cases with a presumed eloquent or semi-eloquent location [25] intraoperative neuromonitoring/awake craniotomy were routinely used.

### Clinical data and follow-up

Pertinent clinical and radiological data were retrieved through a chart review and– if necessary - telephone interviews. All relevant pre- and postoperative oncological treatments as well as cause of death (CNS-disease related or systemic) were documented. Complications were documented using the CTCAE classification framework (Common Terminology Criteria for Adverse Events v5.0; https://ctep.cancer.gov) and recorded in three categories: surgical, medical, and persisting (≥ 30 days following the index surgery) neurological deficits [11].

### Imaging data and volumetric analysis

Preoperative MRI investigations were available for 128 patients (97.7%). 81/131 (61.8%) patients received a postoperative MRI within 72 h of surgery, whereas 45/131 (34.4%) received a CT and 5/131 (3.8%) no early postoperative imaging study. Volumetric analysis was conducted using pre- and postoperative MRI studies and a commercially available software (iplanNet, Brainlab AG, Munich, Germany). We recorded volume of the index metastasis/-es (128 [97.7%] patients), preoperative total tumor load (excluding 4 cases with meningiosis carcinomatosa: 124 [94.7%] patients), and residual total tumor load (i.e. volume of all metastatic disease after removal of the index metastases, excluding the meningiosis cases: 78 [59.5%] patients). All surgeries were also assessed whether they addressed tumors in an eloquent location based on the preoperative imaging studies using the paradigm developed by Sawaya [25].

### Statistical analysis

Commercially available IBM SPSS Statistics for Windows software (Version 25.0, IBM Corp., Armonk, NY) was used for statistical analysis. Kaplan-Meier estimates and log-rank rests were used to study overall survival. Cox and binary logistic regression analyses were performed for multivariate analysis.

## Results

### Patient cohort & tumor characteristics

The series comprised 131 patients (156 surgeries). Median age was 62.0 (IQF 25–75%: 54.0–70.0, range: 32–85) years. Forty-six cases (34.4%) were diagnosed with two metastases. Three and four metastases were present in 22 (14.1%) and 18 (11.5%) cases, respectively (median: 3, IQR 25–75%: 2–6, range: 2–34), i.e. 82 (62.6%) patients had oligometastatic (2–4 tumors) disease [15, 17, 18]. Primary tumors included lung cancer in 67 (51.1%; NSCLC: 51, SCLC: 14, NET/lung neuroendocrine tumor: 2), breast cancer in 26 (19.8%; ER-/PR-/HER2-: 5, ER+/PR+/HER2-: 7, HER2+: 7, N/A: 7), melanoma in 7 (5.3%), colo-rectal carcinoma in 7 (5.3%), and renal cell carcinoma in 4 (3.1%) patients. Five cases (3.8%) were diagnosed with CUP (cancer of unknown primary) syndrome.

Molecular studies of potential targets for therapeutic interventions were performed in 38 patients (breast: 23, lung: 9, melanoma: 2, other: 4). In 3/17 assessable breast cancer cases ER/PR/HER2 status differed between primary tumor and the brain metastasis revealing a new clinically actionable target (i.e. HER2+) [26]. Overall, 14 breast cancer metastases were HER2+. Two NSCLC metastasis tested positive for PD-L-1 which allowed for treatment with pembrolizumab [27].

107/131 (81.2%) patients were followed until death, and median follow-up for patients alive at the time of analysis was 30.3 months (IQR 25–75%: 21.8–43.3). Further characteristics of the cohort can be found in Tables 1 and 2.

**Table 1:** Predictors of functional outcomes (univariate analysis)

		*N*	Discharge KPS 90–100%	*p*-value
Age	> 62 yrs. (median)	69 (52.7%)	26/69 (37.7%)	0.007
	≤ 62 yrs.	62 (47.3%)	38/62 (61.3%)	
Sex	Females	68 (51.9%)	43/68 (63.2%)	< 0.001
	Males	63 (48.1%)	21/63 (32.8%)	
Preoperative (first surgery) KPS	≤ 70%	37 (28.2%)	1/37 (2.7%)	< 0.001
	70–80%	39 (29.8%)	14/39 (35.9%)	
	90–100%	55 (42.0%)	49/55 (89.1%)	
Preoperative seizures	Yes	20 (15.3%)	11/20 (55.0%)	0.550
	No	111 (84.7%)	53/111 (47.7%)	
Extent of CNS disease	Oligometastases (2–4)	82 (62.6%)	43/82 (52.4%)	0.288
	Polymetastases	49 (37.4%)	21/49 (42.9%)	
Tumor load^a^	≤ 18.5 cm^3^	62 (50.0%)	32/62 (51.6%)	0.590
	> 18.5 cm^3^	62 (50.0%)	29/62 (46.8%)	
Index metastasis/-es)^a^	≤ 15.7 cm^3^	64 (50.0%)	30/64 (46.9%)	0.596
	> 15.7 cm^3^	64 (50.0%)	33/64 (51.6%)	
Eloquence (index metastasis/-es; per pat.)	No	93 (71.0%)	38/93 (40.9%)	0.010
	Semi-eloquent	20 (15.3%)	14/20 (70.0%)	
	Eloquent	18 (13.7%)	12/18 (66.7%)	
Histology	Lung	67 (51.1%)	34/67 (50.7%)	0.011
	Breast	26 (19.8%)	18/26 (69.2%)	
	Other	38 (29.0%)	12/38 (32.4%)	
Manifestation	Synchronous	49 (37.4%)	29/49 (59.2%)	0.068
	Metachronous	82 (62.6%)	35/82 (42.7%)	
Extracerebral metastases^b^	Present	87 (69.0%)	42//87 (48.3%)	0.398
	Absent	39 (31.0%)	22/39 (56.4%)	
Perioperative GPA score^b^	1	58 (44.3%)	17/58 (29.3%)	< 0.001
	2	61 (46.6%)	41/61 (67.2%)	
	3 and 4	7 (5.3%)	6/7 (85.7%)	
Residual tumor^c^	≤ 0.28 cm^3^	39 (50.0%)	24/39 (61.5%)	0.173
	> 0.28 cm^3^	39 (50.0%)	18/39 (46.2%)	
Local therapy	All tumors addressed (surgery and/or radiosurgery	40 (30.5%)	20/40 (50.0%)	0.862
	No	91 (69.5%)	44/91 (48.4%)	
New/worsened major neurodeficit	Yes	5 (3.8%)	0	0.058
	No	126 (96.2%)	64/126 (50.8%)	
Major surgical complication	Yes	12 (9.2%)	1/12 (8.3%)	0.004
	No	119 (90.8%)	63/119 (52.9%)	
Major medical complication	Yes	11 (8.4%)	1/11 (9.1%)	0.009
	No	120 (91.6%)	63/120 (52.5%)	

yrs.– years, KPS– Karnofsky performance score, CNS– central nervous system, GPA– graded prognostic assessment [30], ^a^– assessable patients: 124, ^b^– no information: 5 patients, ^c^– assessable patients: 78

**Table 2:** Predictors of overall survival (univariate analysis)

		*N*	mOS (95%CI) in months	*p*-value
Age	> 62 yrs. (median)	69 (52.7%)	10.0 (6.3–13.8)	0.024
	≤ 62 yrs.	62 (47.3%)	4.8 (2.1–7.4)	
Sex	Females	68 (51.9%)	11.3 (4.2–18.4)	0.001
	Males	63 (48.1%)	5.2 (1.0-9.3)	
Preoperative (first surgery) KPS	≤ 70%	37 (28.2%)	2.2 (1.9–2.6)	< 0.001
	70–80%	39 (29.8%)	7.4 (2.3–12.7)	
	90–100%	55 (42.0%)	18.9 (6.4–31.4)	
Postoperative (discharge) KPS	≤ 70%	44 (33.6%)	2.2 (1.7–2.8)	< 0.001
	70–80%	23 (17.6%)	7.4 (1.8–13.0)	
	90–100%	64 (48.9%)	16.5 (8.9–24.1)	
Preoperative seizures	Yes	20 (15.3%)	4.3 (0.0-9.7)	0.831
	No	111 (84.7%)	7.9 (5.5–10.3)	
Extent of CNS disease	Oligometastases (2–4)	82 (62.6%)	10.0 (6.5–13.5)	0.005
	Polymetastases	49 (37.4%)	3.6 (0.5–6.7)	
Tumor load^a^	≤ 18.5 cm^3^	62 (50.0%)	7.3 (5.5–9.1)	0.881
	> 18.5 cm^3^	62 (50.0%)	8.3 (4.3–12.3)	
Index metastasis/-es^a^	≤ 15.7 cm^3^	64 (50.0%)	7.3 (5.1–9.5)	0.854
	> 15.7 cm^3^	64 (50.0%)	8.1 (4.3–11.9)	
Eloquence (index metastasis/-es; per pat.)	No	93 (71.0%)	7.3 (4.7–9.9)	0.168
	Semi-eloquent	20 (15.3%)	9.2 82.7–15.7)	
	Eloquent	18 (13.7%)	6.7 (1.5–11.8)	
Histology	Lung	67 (51.1%)	7.4 (5.3–9.5)	0.003
	Breast	26 (19.8%)	67.2 (0.0-146.1)	
	Other	38 (29.0%)	5.4 (0.9–10.0)	
Manifestation	Synchronous	49 (37.4%)	7.5 (5.4–9.6)	0.866
	Metachronous	82 (62.6%)	7.3 (3.8–10.8)	
Extracerebral metastases^b^	Present	87 (69.0%)	7.5 (4.4–10.6)	0.606
	Absent	39 (31.0%)	8.3 (4.6–11.9)	
Perioperative GPA score^b^	1	58 (44.3%)	3.1 (0.8–5.5)	0.009
	2	61 (46.6%)	10.5 (7.5–13.5)	
	3 and 4	7 (5.3%)	67.2 (0.0-136.4)	
Residual tumor^c^	≤ 0.28 cm^3^ > 0.28 cm^3^	39 (50.0%) 39 (50.0%)	5.8 (1.5–10.1%) 10.7 (7.4–13.9)	0.012
Local therapy	All tumors addressed (surgery and/or radiosurgery	40 (30.5%)	8.1 (5.7–10.4)	0.189
	No	91 (69.5%)	6.7 (4.0-9.3)	
New/worsened major neurodeficit	Yes	5 (3.8%)	0.3 (0-0.8)	< 0.001
	No	126 (96.2%)	7.6 (5.4–9.7)	
Major surgical complication	Yes	12 (9.2%)	2.3 (0-12.3)	0.506
	No	119 (90.8%)	7.4 (4.9–10.0)	
Major medical complication	Yes	11 (8.4%)	2.3 (1.3–3.3)	0.017
	No	120 (91.6%)	8.1 (6.0-10.1)	
Postoperative RT^d^	Yes	94 (73.2%)	11.3 (7.4–15.6)	< 0.001
	No	36 (27.7%)	1.5 (1.0–2.0)	
Postoperative ST^e^	Yes	76 (59.8%)	14.8 (9.6–19.9)	< 0.001
	No	51 (40.2%)	2.0 (1.6–2.4)	

mOS - median overall survival (months), 95%CI– 95% confidence interval, yrs.– years, KPS– Karnofsky performance score, CNS– central nervous system, GPA– graded prognostic assessment [30], RT– fractionated radiotherapy, ST– systemic tumor therapy, ^a^– assessable patients: 124, ^b^– no information: 5 patients, ^c^– assessable patients: 78, ^d^– no information: 1, ^e^– no information. 4

### Surgical treatment & adjuvant therapies

**Fig. 1:**
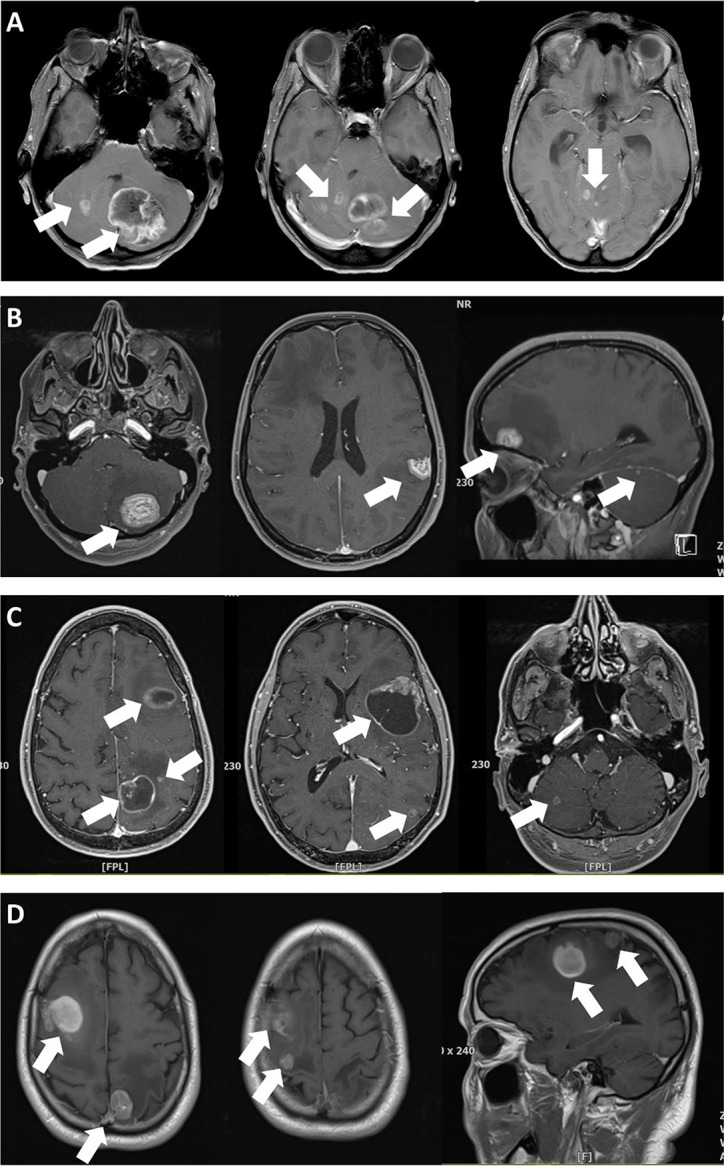
Examples of surgical indications (T1 weighted & contrast-enhanced MR images) **a** 51-year-old female patient with multiple brain metastases in the posterior fossa (arrows) and known esophageal cancer. Surgery was performed primarily in order to avoid occlusive hydrocephalus, i.e. only the large left mass consisting of several coalescing metastases was removed. The patient survived for 34 months following the operation. **b** 35-year-old female with breast cancer (ER+, PR+, HER2+), hepatic metastases diagnosed 41 months earlier and four brain metastases (arrows). She presented with headaches and a visual field cut. The treating oncologist requested tissue for molecular analyses (“molecularly informed treatment”). We felt that this case might benefit from aggressive local treatment of all CNS cancer manifestations. Hence, the patient had two surgeries for removal (1) of the left cerebellar and temporo-parietal metastases and (2) the right fronto-basal tumor. All tumor were HER+, but ER-/PR-. The small right cerebellar metastasis was treated with radiosurgery. We favored surgery for the frontal and the temporo-parietal lesions in order to save radiosurgery as an option for tumor recurrence. Indeed, despite adjuvant radiotherapy further radiosurgeries and even one additional surgery for a left cerebellar recurrence were required later on for CNS disease control. The patient was alive and well at her last follow-up 30 months after the initial operations. **C** 58-year-old male patient presenting with multiple brain metastases (arrows; additional small tumors in the left temporal and both occipital lobes), mild aphasia and no known primary tumor. Initial staging investigations suggested lung cancer as the primary tumor. However, a bronchoscopy failed to establish a tissue diagnosis. Awake surgery was performed for removal of the left frontal lesion in order to relieve the mass effect and its consecutive neurological symptoms (aphasia) and to obtain a tissue diagnosis. The patient improved and was neurologically intact after the surgery. Pathological evaluation of the CNS metastasis revealed carcinoma with neuroendocrine differentiation well in line with the presumptive diagnosis of primary lung cancer. **d** 66-year-old male patient diagnosed with malignant melanoma 10 years earlier and presenting with moderate paresis of the right hand > arm. The tumors (arrows) were removed through two separate craniotomies with the help of intraoperative MEP neuromonitoring. Surgery was favored over primary radiotherapy/-surgery because of the presumed resistance of malignant melanoma CNS metastases to radiotherapy and in an attempt to preserve the patient’s neurological status though removal of the lesion in the right precentral gyrus. The patient’s neurodeficit indeed improved slightly after surgery

Twenty-three patients underwent two and two patients had three operations. One patient underwent a re-resection of residual tumor. Patients often had more than one indication to undergo surgery. Surgical indications included mass effect (posterior fossa: 62, supratentorial disease: 68, both: 2), no known primary cancer (50), history of multiple cancers (12), known radioresistant primary tumor (7), and tumor progress after radiosurgery/-therapy (3). In 10 patients with a known primary tumor, tissue acquisition was nevertheless requested by the treating oncologist specifically for additional molecular pathological studies. Examples of surgical indications are shown in Fig. 1. Neuromonitoring and/or awake craniotomy were used in 38 surgeries. In 40 cases (30.5%) all metastatic deposits were addressed by local treatments (i.e. surgery: 29 [22.1%], surgery & radiosurgery: 11 [8.4%]).

Postoperative radiotherapy was administered in 94/130 (72.3%) cases and systemic therapy in 76/127 (59.8%) patients. Thirty-one (24.4%) of 127 cases had no adjuvant treatments.

### Complications & functional outcomes

Major (i.e. CTCAE grade 3–5) neurological, surgical and medical complication were observed in 6 (3.8%), 12 (7.7%), and 12 (7.7%) surgical cases. Interestingly, the neurodeficit rates did not vary significantly with the eloquence of the tumor location (eloquent: 0/19 [0%], semi-eloquent: 1/24 [4.2%], non-eloquent: 5/113 [4.4%], p = NS).

Median discharge KPS was 80% (IQF: 60–90%, range 0-100%). Following surgery, the KPS improved in 38/131 (29.0%). A worsening of the KPS was seen in 29/131 (22.1%; ≥ 20: 16 [12.2%]). Univariate predictors of a good functional outcome (KPS 90–100%) included younger age, female sex, better preoperative KPS, (semi)-eloquent location of the index metastasis/-es, histology (best functional outcomes in breast cancer cases), and no major surgical or medical complication (Table 1**)**. A multivariate analysis with all variables which correlated significantly with better KPS outcomes in the univarate analysis showed statistically significant associations for a higher preoperative KPS, no major postoperative complication, and a (semi)-eloquent tumor location (Table 3).

**Table 3:** Predictors of a good (KPS 90–100%) functional outcome at discharge (multivariate analysis)

		RR	95% CI	*p*-value
Age	> 62 yrs. (median)	0.388	0.122–1.234	0.109
Sex	female	3.305	0.900-12.393	0.072
Preoperative (first surgery) KPS	≤ 80% (vs. 90–100%)	0.017	0.004–0.071	< 0.001
Histology^a^				0.591
	lung	1.994	0.524–7.594	0.312
	breast	1.701	0.292–9.907	0.555
Eloquence	(Semi)-eloquent vs. no	3.839	1.037–14.205	0.044
Any major complication^b^	Neurodeficit, surgical and/or medical	0.042	0.005–0.376	0.005

95%CI– 95% confidence interval, yrs.– years, KPS– Karnofsky performance score, ^a^– reference category, ^b^– CTCAE (common terminology criteria for adverse events) grade 3–5

Of note, patients with a better discharge KPS had significantly more often systemic treatments (KPS 90–100: 47/53 [88.7%] vs. KPS ≤ 80: 29/74 [39.2%], *p* < 0.001) and/or radiotherapy (KPS 90–100: 51/54 [94.4%] vs. KPS ≤ 80: 43/76 [56.6%], *p* < 0.001) after their operation. 31 cases (23.8%, unknown: 1) had no postoperative systemic or radiotherapy. 22/31 (71.0%) of these cases had a KPS of 60% or worse, i.e. an adverse functional health status was the major reason for not undergoing postoperative treatment. None of the cases with a new/worsened major neurodeficit had radiotherapy (*p* < 0.001) or adjuvant systemic treatment (*p* = 0.009). Surgical and medical complications also correlated inversely with postoperative treatment; however, these findings were statistically significant only for medical complications and systemic therapies.

### Patient survival

Median overall survival (mOS) was 7.4 months (95% CI: 5.4–9.5). Estimated 1 year overall survival rate was 35.6%, and the 2 year rate was 25.1%. 30-day-mortality was 10.7% (14/131) including 10 (7.6%) patients who died from complications of the primary disease unrelated to the index surgery. Cause of death was available for 106 (80.9%) patients. 25/106 (23.6%) died due to CNS disease.

Positive univariate predictors of overall survival included younger age (≤ 62 years), female sex, higher pre- (day 1 before surgery) and postoperative KPS (i.e. at discharge, assessed at 5 days (median, IQR 25–75%: 6.0–14.0) after surgery) and oligometastatic (2–4 tumors) disease. Patients with oligometastases had significantly higher pre- and postoperative KPS, and significantly lower residual tumors volumes (Supplementary Table 1). Survival also varied strongly with primary tumor histology, e.g. mOS in patients with breast cancer was 67.2 months, and the estimated 3-year overall survival rate was 55.6%. The presence of extracerebral metastases did not impact on OS. Survival was dismal (i.e. mOS ≤ 2.5 months) in patients who had no postoperative radio- and systemic therapy, or who incurred major complications (see Table 2). We investigated the role of postoperative treatment separately in patients with lung cancer, i.e. the largest subgroup defined by their primary cancer. Patients who received postoperative radio- and systemic treatment had a significant better overall survival: (mOS, surgery only: 0.9 months (95% CI 0.2–1.6) vs. surgery and postoperative monotherapy: 3.2 months (2.4-4.0) vs. surgery and radio- as well as systemic therapy: 13.6 months (8.0-19.2); *p* < 0.001)).

**Fig. 2:**
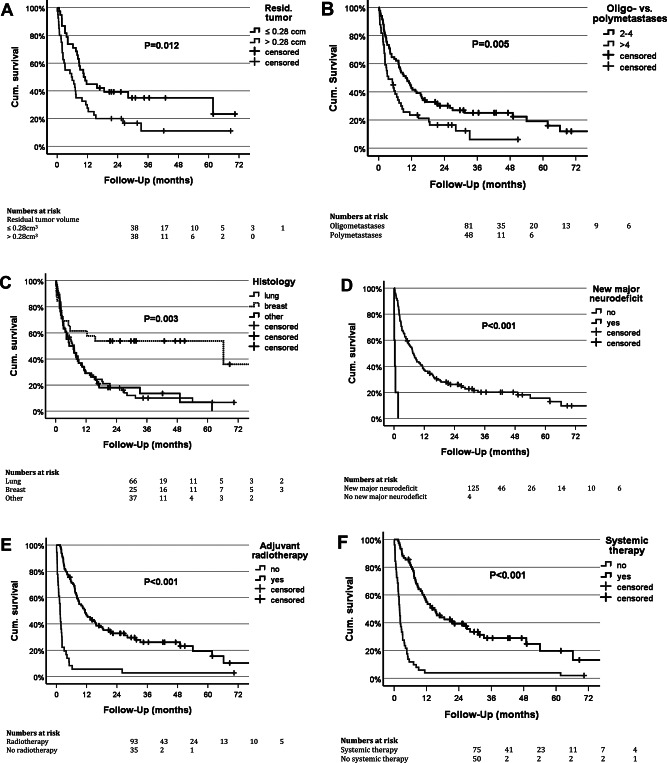
Impact of selected prognostic parameters on overall survival (Kaplan Meier estimates) **a** Extent of resection. A survival benefit is associated with resections resulting in ≤ 0.28 cm^3^ residual tumor (≈ corresponding to a spherical lesion with a 0.8 cm diameter) **b** Extent of CNS disease. Survival is better in patients with oligometastases (2–4) vs. polymetastases (> 4). **c** Primary tumor histology. The relatively best survival was seen in patients with breast cancer. **d** New/worsend major (CTCAE grade 3–5) neurodeficit. Neurological worsening is associated with a dismal prognosis. **e** Postoperative radiotherapy & **f** Postoperative systemic therapy. Overall survival of cases not undergoing postoperative radiotherapy and/or systemic treatment is very poor reflecting the importance of local CNS as well as systemic disease control but also the role of maintaining/improving the functional status in order to enable the patient to undergo postoperative treatments

Preoperative total tumor load did not influence OS. Likewise, the volume of the index metastases was not prognostic. Aggressive local therapy (surgery and/or radiosurgery) for all brain metastases did not carry a survival benefit in our cohort. However, extent of resection was prognostic. Using median residual tumor volume (0.28 cm^3^) as the cut-off, patients with less residual tumor had a significantly better mOS (10.7 months, cf. 5.8 months for cases with > 0.28 cm^3^ residual tumor; *p* = 0.012; Table 2; Fig. 2).

Multivariate analysis with all parameters significantly correlated with survival as univariate parameters revealed female sex, oligometastatic (2–4 tumors) disease, no major new/worsened neurodeficit, and postoperative radio- and systemic therapy as independent positive prognostic parameters in our cohort (Table 4).

**Table 4:** Predictors of overall survival (multivariate analysis)

		HR	95% CI	*p*-value
Age (per yr.)	> 62 yrs. (median)	1.151	0.722–1.836	0.554
Sex	female	0.590	0.361–0.966	0.036
Preoperative (first surgery) KPS	≤ 80% (vs. 90–100%)	1.561	0.940–2.594	0.085
Histology^a^				0.741
	lung	0.951	0.599–1.510	0.832
	breast	0.744	0.349–1.586	0.444
Extent of CNS disease	> 4 metastases. (vs. oligometastases: 2–4)	2.280	1.492–3.485	< 0.001
New/worsened major neurodeficit^b^	yes	9.212	3.089–27.470	< 0.001
Major medical complication)^b^	yes	1.493	0.710–3.140	0.290
Postoperative RT	yes	0.439	0.250–0.769	0.004
Postoperative ST	yes	0.370	0.207–0.661	< 0.001

95%CI– 95% confidence interval, yrs.– years, KPS– Karnofsky performance score, CNS– central nervous system, RT– fractionated radiotherapy, ST– systemic tumor therapy, ^a^– reference category, ^b^– CTCAE (common terminology criteria for adverse events) grade 3–5

## Discussion

For the present paper we have reviewed our 2015–2021 institutional experience with the surgical treatment of patients with multiple brain metastases. Similar to what has been reported in the literature [28–30], survival in our cohort was limited (median overall survival: 7.4 months), however, varied greatly between patients. Comparing the results reported in surgical series with the radiotherapy [31, 32] and radiosurgery literature [33] is very difficult due to the inherent heavy patient selection bias. A very sizable percentage of the patients with multiple brain metastases will survive beyond 1–2 years. Of note, most patients (including our own) described in surgical multiple brain metastases series harbor only 2 to 4 metastases, i.e. suffer from oligometastases [15, 17, 18]. Our data suggest a prominent role for the extent of the CNS disease (assessed by number of brain metastases) as a prognostic factor and thereby support the concept of CNS oligometastases.

Operating patients in order to obtain tissue (diagnoses) appears to play a most important role in the management of cases with multiple brain metastases. For instance, surgical indications in our cohort included no known primary cancer in as many as 38.2% and a history of multiple cancers in 9.2%. Surgery was performed in 7.6% of our cases in order to access tissue for biomarker/target analysis. This is a relatively new indication and may well be performed increasingly more often in the future [34]. Patients with breast cancer from this series had a much better prognosis than the remainder of our cohort, which would also explain in part superior survival seen in females. Explanations of this finding certainly include selection bias and the very limited number of cases analyzed. The relative percentage of HER2+ (7/19 [36.8%]) but also of prognostically unfavorable triple-negative (ER-/PR-/HER2-) cases was similar to a recently reported large cohort of breast cancer patients with CNS metastases [35]. Interestingly, Sperduto et al. [3] described overall improving survival in patients with brain metastases in general, but in particular in breast cancer cases. Contradictory findings have also been reported [8].

The strong impact of postoperative radiotherapy and systemic treatment on survival highlights the paramount importance of both local (CNS) as well as systemic disease control. Postoperative irradiation is an integral part of most therapeutic concepts aiming at CNS disease control [36, 37]. Our data indicate that the primary role of surgery in patients with multiple brain metastases is to enable them to undergo further oncological treatment. Most agents used for systemic therapies clinically do not work for brain metastases. Hence, the major prognostic role of systemic tumor therapy likely reflects the importance of treating the systemic cancer once the CNS disease is controlled. Only approximately one quarter of our cases eventually died from CNS disease. This figure suggests that current local therapeutic concepts for multiple brain metastases work reasonably well, so that future advances in systemic oncological therapy may hopefully translate into significantly improved survival in these patients.

Complications and an adverse functional health status very often preclude postoperative treatments which at least in part explains their negative prognostic impact. In our cohort, new persistent CTCAE grade 3–5 neurological deficits were seen in 3.8%, the corresponding figures for surgical and medical complications were 7.7% and 7.7%, respectively. Most of our patients retained their preoperative KPS. An improved KPS was seen in 38/131 (29.0%), but also KPS worsening (e.g. 22.1% KPS drop ≥ 20) in a sizable number of cases. Similar figures (including the sizable proportion of cases with functional improvement following surgery) have also been published by others [3, 5–7, 9, 28, 38].

Surgical strategies therefore have to pay much attention to complication avoidance [7, 11]. (Piecemeal) surgical removal and opening of the ventricular system have been discussed as potential risk factors for secondary leptomeningeal dissemination (LMD) after surgery [39, 40]. We routinely use techniques and adjuncts such as intraoperative monitoring and awake craniotomies (Fig. 1) aiming at preserving neurological function similar as in operations for intrinsic tumors. Interestingly, eloquence of the tumor location did not negatively impact on neurodeficit rates in this series. Functional outcomes were even better in patients with surgery for (semi)-eloquent tumors. Besides selection bias, this may in part reflect the role of surgery in preserving or even improving function. At least these data suggest that an eloquent location of brain metastases should not deter from surgical treatment.

The most controversial indication for surgery in cases with multiple brain metastases may well be surgical cytoreduction beyond relieving mass effect [12–14, 41]. Less than median residual metastatic disease volume (= 0.28 cm^3^) correlated significantly with better survival in the present series. However, this effect was lost in the multivariate analysis. A 0.28 cm^3^ tumor residual would correspond to a spheric lesion with a diameter of 0.8 cm. This figure is very similar to the 1 cm cut-off used to distinguish between measurable and non-measurable disease in the RANO criteria [42]. Hence, our data may indicate that surgical cytoreduction aiming at removing all “measurable” disease may carry a survival benefit. It should be noted, however, that our data somewhat contradictorily did not confirm that a policy of addressing all brain metastases with surgery and/or radiosurgery carries a survival benefit [3, 23, 24, 43].

Our study has significant limitations. Albeit studying consecutive patients, data acquisition and analysis was performed retrospectively. Volumetric analyses were not available for all patients. Many patients received their postoperative treatment in outside institutions. Finally, our analysis cannot account for the very significant selection bias that not all patients with multiple brain metastases are referred for surgical treatment or at least a neurosurgical opinion.

Nevertheless, taken together, our investigation provides robust data to suggest that patients with multiple brain metastases can benefit from surgery most likely because surgical tumor removal reduces mass effect and thereby often helps to maintain or even improve a patient’ functional status which creates the necessary time for effective radio- and medical oncological therapy [6, 41].

## Conclusion

Surgery is a reasonable therapeutic option in many patients with multiple brain metastases despite their overall poor prognosis. Diagnostic surgery for the acquisition of metastatic tissue (more recently also to help with the identification of potentially “drugable” targets) is an important surgical indication. Operations should primarily aim at preserving or even improving the patients’ functional health status in order to enable them to undergo further local (usually radiation) and systemic therapy. The surgical goal is a maximum safe cytoreduction (which may carry a survival benefit by itself) and therefore may require the routine use of neuromonitoring and/or awake craniotomy.

### Electronic supplementary material

Below is the link to the electronic supplementary material.

Supplementary Material 1

## Data Availability

No datasets were generated or analysed during the current study.
