# Supplementary material for: Defining the role of surgery for patients with multiple brain metastases
Source: J Neurooncol. 2024 Jun 25;169(2):317–28. doi: 10.1007/s11060-024-04739-7 (PMC11341766; doi:10.1007/s11060-024-04739-7)
Supplement: Supplementary file 1 — Supplementary Material 1 [file 11060_2024_4739_MOESM1_ESM.docx]

**Supplemtary Table 1:** Characteristics of patients’ with oligometastases

|  |  | ***Oligometastases*** | ***Polymetastases*** | ***p-value*** |
| --- | --- | --- | --- | --- |
| **Age** | > 62 yrs. (median) | 40/80 (50.0%) | 29/49 (59.2%) | 0.249 |
|  | ≤ 62 yrs. | 42/82 (50.0%) | 20/49 (40.8%) |  |
| **Sex** | Females | 43/82 (52.4%) | 25/68 (36.8%) | 0.875 |
|  | Males | 39/82 (47.6%) | 24/63 (63.2%) |  |
| **Preoperative (first surgery) KPS** | ≤ 70% | 18/82 (22.0%) | 21/49 (42.9%) | 0.028 |
|  | 70-80% | 27/82 (32.9%) | 9/49 (18.4%) |  |
|  | 90-100% | 37/82 (45.1%) | 19/49 (38.8%) |  |
| **Postoperative (at discharge) KPS** | ≤ 70% | 14/64 (21.9%) | 21/45 (46.7%) | 0.021 |
|  | 70-80% | 18/64 (28.1%) | 7/45 (15.6%) |  |
|  | 90-100% | 32/64 (50.0%) | 17/45 (37.8%) |  |
| **Preoperative seizures** | Yes | 11/82 (13.4%) | 9/49 (18.4%) | 0.446 |
|  | No | 71/82 (86.6%) | 40/49 (81.6%) |  |
| **Postoperative seizures** | Yes | 4/82 (8.9%) | 1/49 (2.0%) | 0.412 |
|  | No | 78/82 (95.1%) | 48/49 (98.0%) |  |
| **Tumor load^a^** | ≤ 18.5 cm^3^ | 40/79 (50.6%) | 23/45 (51.1%) | 0.852 |
|  | > 18.5 cm^3^ | 39/79 (49.4%) | 22/45 (48.9%) |  |
| **Index metastasis/-es)^a^** | ≤ 15.7 cm^3^ | 35/79 (44.3%) | 29/49 (59.2%) | 0.102 |
|  | > 15.7 cm^3^ | 44/79 (55.7%) | 20/49 (40.8%) |  |
| **Eloquence (index metastasis/-es; per pat.)** | No | 57/82 (69.5%) | 42/49 (85.7%) | 0.098 |
|  | Semi-eloquent | 14/82 (17.1%) | 3/49 (6.1%) |  |
|  | Eloquent | 11/82 (13.4%) | 4/49 (8.2%) |  |
| **Histology** | Lung | 39/82 (47.6%) | 28/49 (57.1%) | 0.087 |
|  | Breast | 18/82 (21.9%) | 8/49 (16.3%) |  |
|  | Other | 25/82 (30.5%) | 13/49 (26.5%) |  |
| **Manifestation** | Synchronous | 55/82 (67.1%) | 27/49 (55.1%) | 0.171 |
|  | Metachronous | 27/82 (32.9%) | 22/49 (44.9%) |  |
| **Extracerebral metastases^b^** | Present | 56/80 (70.0%) | 31/46 (67.4%) | 0.760 |
|  | Absent | 24/80 (30.0%) | 15/46 (32.6%) |  |
| **Perioperative GPA score^b^** | 1 | 29/80 (36.3%) | 29/46 (63.0%) | 0.005 |
|  | 2 | 44/80 (55.0%) | 17/46 (37.0%) |  |
|  | 3 and 4 | 7/80 (8.7%) | 0/7 (0.0%) |  |
| **Residual tumor^c^** | ≤ 0.28 cm^3^ | 35/38 (92.1%) | 15/40 (37.5%) | < 0.0001 |
|  | > 0.28 cm^3^ | 3/38 (7.9%) | 25/40 (62.5%) |  |
| **Local therapy** | All tumors addressed (surgery and/or radiosurgery | 40/82 (48.8%) | 0/49 (0.0%) | < 0.0001 |
|  | No | 42/82 (51.2%) | 49/49 (100.0 %) |  |
| **New/worsened major neurodeficit** | Yes | 3/82 (3.7%) | 2/49 (4.1%) | 0.903 |
|  | No | 79/82 (96.3%) | 47/49 (95.9%) |  |
| **Major surgical complication** | Yes | 8/82 (9.8%) | 4/49 (8.2%) | 0.760 |
|  | No | 74/82 (90.2%) | 45/49 (91.8%) |  |
| **Major medical complication** | Yes | 6/82 (7.3%) | 5/49 (10.2%) | 0.564 |
|  | No | 76/82 (92.7%) | 44/49 (89.8%) |  |
| **Postoperative radiotherapy^d^** | Yes | 61/81 (75.3%) | 33/49 (67.3%) | 0.326 |
|  | No | 20/81 (24.7%) | 16/49 (32.7%) |  |
| **Postoperative systemic therapy^e^** | Yes | 52/80 (65.0%) | 24/47 (51.1%) | 0.122 |
|  | No | 28/80 (35.0%) | 23/47 (48.9%) |  |

yrs. – years, KPS – Karnofsky performance score, GPA – graded prognostic assessment [30], ^a^ – assessable patients: 124, ^b^ – no information: 5 patients, ^c^ – assessable patients: 78, ^d^ – no information: 1, ^e^ – no information 4.
